# A Community-based Cervical Cancer Screening Program among Women of Delhi using Camp Approach

**DOI:** 10.4103/0970-0218.62576

**Published:** 2010-01

**Authors:** Pragya Sharma, Manju Rahi, Panna Lal

**Affiliations:** Public Health Specialist, NRHM, GNCTD, New Delhi - 110 002, India; 1 Department of Community Medicine, Maulana Azad Medical College, New Delhi - 110 002, India; 2 Indian Council of Medical Research (ICMR), New Delhi - 110 002, India

**Keywords:** Camp approach, cancer cervix, community-based, screening

## Abstract

**Background:**

Cervical cancer is the commonest malignancy among women in developing countries. Cytological screening (Pap smear) have been claimed to reduce incidence and mortality of carcinoma cervix significantly for which sensitization of women is required through community-based approach.

**Objectives:**

To find out number of cervical cancer cases among patients reporting to a general health care camp through screening program and study the prevalence of perceived morbidity and its confirmation.

**Settings:**

Cross-sectional study among women attending cancer awareness camps.

**Materials and Methods:**

A total of 435 women attending cancer awareness camps were screened for carcinoma cervix. The findings of history and clinical examination were recorded. Pap smears of all the symptomatic patients were collected and cytological diagnosis was confirmed by a pathologist.

**Results and Conclusions:**

The perceived gynecological morbidity was observed to be 59.8%. The smear of the women who were suspected of carcinoma on clinical examination was confirmed to be the cases of carcinoma-*in-situ* (7.8%) and high-grade neoplasia (2.9%) on laboratory investigations. The findings of the study highlight the utility and need of cancer cervix screening among the women at regular intervals through camp approach in the community.

## Introduction

Cervical cancer is the commonest malignancy among women in India(1) and second most common form of cancer in the world as a whole. Worldwide, particularly in developing countries, cervical cancer remains the major cause of death in women accounting for an estimated 160 000 deaths every year.(2) The age adjusted incidence rate for cervical cancer has been reported to vary from 19 to 44/100 000 women in various cancer registries in India.(3)

For the prevention by early diagnosis of cervical cancer, only one proven strategy currently available is cytological screening (Pap smear). Many studies have shown that carcinoma *in situ* can be detected for several years before it progresses to invasive cancer by cytological screening.(4) The screening programs every five years in several countries have been able to reduce the incidence and mortality from cervical cancer by 60% as observed by Hakama *et al.*(2,4) Thus there is a need to sensitize women through awareness campaigns to undergo cervical screening at regular intervals. The present study was undertaken with the objectives of: 1) To find out number of cervical cancer suspects/cases among patients reporting to a general health care camp through screening program and 2) To study the prevalence of perceived morbidity and its confirmation among them.

## Materials and Methods

This cross-sectional study has been carried out in field practice areas attached to the department of community medicine, Maulana Azad Medical College. The cancer awareness camps were organized at an interval of one week during the period between October 31 and December 11, 2003, at the four centers namely Primary School Ganga Vihar, Urban Health Center Gokulpuri, Basti Vikas Kendra Vikram Nagar and Sarva Shiksha Abhiyan Kendra, Jasola. Each camp was visited by a team of specialist doctors consisting of a gynecologist, surgeon and a physician from Lok Nayak Jai Prakash Hospital (LNJP) hospital, New Delhi. A total of 894 patients including 459 males and 435 females attended these camps. Of them, among females, 214 patients had at least one complaint regarding reproductive tract morbidity. All of them were examined clinically. The findings of history and clinical examination were recorded on the proforma provided by Institute of Cytology and Preventive Oncology (ICPO). Pap smears of all the symptomatic patients were collected. The cytological diagnosis was confirmed by a pathologist by examining the pap smears. The data was analysed by calculating percentages.

## Results and Discussion

### Sociodemographic characteristics

Majority (95.7%) of the women attending the camp were in the reproductive age group (15-44 years) and illiterate (64.4%). It was observed that 53.3% of women had at least one but less than three children. Most (82.53%) of the women attending the camp had a family income of more than 1000 rupees per month. About 37.9% women had more than three children born any time in their reproductive period [Table 1]. The findings of the present study correspond with the results obtained from a study conducted in similar settings.(1)

**Table 1:** Sociodemographic profile of females attending the camp

Characteristics	No. of subjects (*n* = 435)	%
Age (years)		
15-24	176	40.5
25-34	160	36.8
35-44	80	18.4
45-54	16	3.7
55-64	8	1.8
>65	15	3.4
Education status		
Illiterate	280	64.4
Primary	104	23.9
Matric	36	8.2
Senior secondary	12	2.7
Graduate and above	3	0.7
Income (Rs. p.m.)		
≤1000	76	17.5
>1000	359	82.5
Parity		
Nil	38	8.7
1-3	232	53.3
>3	165	37.9

### Types of perceived morbidity

A total of 214 (59.8%) women attending the camp had one or the other symptoms of reproductive tract morbidity. Discharge per vaginum and pain in lower abdomen were the most common complaints reported by 28.5 and 20.1% women, respectively. This can be attributed to poor genital hygiene and presence of sexually transmitted diseases (STDs).(1) Some of the STDs may also act as precursors for cancer cervix, thus may require prompt treatment as reported in other studies also.(4) Other complaints reported by the study subjects were backache (16.2%), menstrual abnormalities (15.6%) and dysuria (7.5%). A few of the women had complaints of dyspareunia (2.75%) and postmenopausal bleeding (1.4%) [Table 2].

**Table 2:** Type of perceived reproductive tract morbidity

Symptom	No. of patients	Percentages
Discharge per vaginum	102	28.5
Pain lower abdomen	72	20.1
Backache	58	16.2
Menstrual abnormalities	56	15.6
Dysuria	27	7.5
Pruritus vulvae	20	5.5
Dyspareunia	10	2.8
Infertility	08	2.2
Postmenopausal bleeding	05	1.4
Total no. of complaints	358*	100
Any morbidity	214	59.8

* Some patients had more than one complaint

### Clinical diagnosis

Among 214 women examined clinically, cervical erosion (22%), cervicitis (13.1%), vaginitis (8.4%) and cervical hypertrophy (7.9%) were the most common pathological conditions observed. Suspicious malignancy and atrophy of cervix were found in 4.2 and 8.4% of patients, respectively. Most of the patients, who complained of any morbidity, were married at an age below 18 years, had borne more than three children and were illiterate. The percentage of cases diagnosed to be suffering from one or the other reproductive morbidity decreased with increasing age at marriage and literacy levels and also with decreasing parity. Among risk factors associated with morbidity among these women, age at marriage less than 18 years (31.45%), high parity (30.56%) and illiteracy leading to poor genital hygiene (41%) were observed to be the prominent risk factors. Some other studies have also reported a significant association of cancer cervix with these risk factors.(5–7)

A significant association of cervical carcinoma was observed in the present study with the age at marriage and number of children born to a woman during her reproductive life and their literacy levels. The prevalence of carcinoma *in situ* and high grade cervical carcinoma was found collectively to be 9.4% in women married at less than 18 years of age. The estimated relative risk for cancer cervix among women getting married before 17 years of age has been reported to be 7.9 in a study conducted by Dutta.(5,7) The prevalence of carcinoma *in situ* and high grade cervical carcinoma was found out to be 10.7% amongst women with high parity (more than three children). Similar to the observations in the present study, a significant association between high parity and cervical cancer has been reported with a relative risk of two in other studies also(5,1) [Tables 3 and 4].

**Table 3:** Relation of clinical findings with age at marriage, parity and education

Clinical diagnosis	Age at marriage (years)			Parity			Education	
			
	<18 (*n* = 106)	18-25 (*n* = 84)	>25 (*n* = 24)	≤1 (*n* = 21)	1-3 (*n* = 90)	>3 (*n* = 103)	Illiterate (*n* = 170)	Literate (*n* = 44)
Erosion	22 (20.8)	17 (20.2)	04 (16.7)	02 (9.5)	19 (21.1)	22 (21.4)	34 (20)	09 (20.5)
Cervicitis	13 (12.3)	11 (13.2)	04 (16.7)	0 (0.0)	16 (17.8)	12 (11.6)	23 (13.5)	05 (11.4)
Hypertrophy cervix	09 (8.5)	06 (7.1)	02 (8.4)	01 (4.8)	06 (6.7)	10 (9.7)	15 (8.8)	02 (4.5)
Vaginitis	11 (10.4)	06 (7.1)	01 (4.2)	02 (9.5)	05 (5.6)	11 (10.7)	12 (7.1)	06 (13.6)
Polyp	12 (11.3)	08 (9.5)	02 (8.4)	02 (9.5)	10 (11.1)	08 (7.8)	15 (8.8)	04 (9.1)
Atrophic cervix	10 (9.4)	06 (7.1)	02 (8.4)	02 (9.5)	04 (4.4)	12 (12.4)	15 (8.8)	03 (6.8)
DUB/PID	20 (18.9)	15 (17.9)	05 (20.8)	05 (23.8)	17 (18.9)	18 (17.5)	35 (20.6)	5 (11.4)
Cervix firm to hard	02 (1.9)	07 (8.3)	02 (8.4)	05 (23.8)	04 (4.4)	02 (1.9)	07 (4.1)	04 (9.1)
Prolapse	01 (9.5)	06 (7.1)	01 (4.2)	01 (4.8)	05 (5.6)	02 (1.9)	05 (2.9)	03 (6.8)
Suspicious for malignancy	06 (5.7)	02 (2.4)	01 (4.2)	01 (4.8)	04 (4.4)	04 (3.9)	09 (5.3)	03 (6.8)

**Table 4:** Relation of Pap smear findings with age at marriage, parity and education

Lab diagnosis	Age at marriage (years)			Parity			Education	
			
	<18 (*n* = 106)	18-25 (*n* = 84)	>25 (*n* = 24)	≤1 (*n* = 21)	1-3 (*n* = 90)	>3 (*n* = 103)	Illiterate (*n* = 170)	Literate (*n* = 44)
Benign inflammatory change	45 (42.4)	35 (41.6)	08 (33.3)	10 (47.6)	34 (37.8)	44 (42.7)	70 (41)	18 (40.9)
Infection-*G.vaginalis*	20 (18.8)	24 (28.5)	06 (25)	03 (14.3)	22 (24.4)	25 (24.3)	40 (23.5)	10 (22.7)
Normal	15 (14.1)	12 (14.3)	07 (29.2)	05 (23.8)	19 (21.1)	10 (9.7)	30 (17.6)	04 (9.1)
Atrophic endometrium	10 (9.4)	06 (7.1)	01 (4.2)	01 (4.8)	07 (7.8)	09 (8.7)	13 (7.6)	04 (9.1)
Unsatisfactory sample	06 (5.6)	05 (5.9)	01 (4.2)	02 (9.5)	06 (6.7)	04 (3.9)	07 (4.1)	05 (11.4)
Carcinoma *in situ*	07 (6.6)	02 (2.4)	01 (4.2)	0 (0.0)	02 (2.2)	08 (7.8)	07 (4.1)	03 (6.8)
High grade carcinoma	03 (2.8)	0 (0.0)	0 (0.0)	0 (0.0)	0 (0.0)	03 (2.9)	03 (1.8)	0 (0.0)

### Laboratory diagnosis

The pap smear slides were collected from all 214 women for the confirmation of diagnoses. Of the smears, 41.1% smears showed benign inflammatory changes and 23.4% had an evidence of infection, 15.9% were normal whereas 5.6% samples were found to be unsatisfactory. As many as 7.9% slides showed atrophic endometrium whereas 4.67% showed carcinoma *in situ* and 1.4% showed high grade malignancy. However, smear of the women who had suspicion of carcinoma on clinical examination confirmed to be the cases of carcinoma-in-situ (7.8%) and high-grade neoplasia (2.9%) [Table 4].

Carcinoma-*in-situ* is the stage which could be reversed by early treatment, and mortality due to high grade carcinoma thus could be reduced to a considerable extent as has been observed by others.(2,3) Thus such types of screening camps and programs must be held more frequently in order to curb the menace due to cervical cancer.

## References

[1] Desai M (2004). An assessment of community based cancer screening program among Indian Women Using the Anganwadi Workers. J obstet Gynecol Ind.

[2] Hakama M, Miller AB, Day NE, IARC (1987). Working Group on Cervical cancer screening. Screening for Cancer of the Uterine Cervix.

[3] National Cancer Registry Programme (1984-1993). Ten year Consolidated report of the Hospital Based Cancer Registries.

[4] World Health Organization (2006). Cervical Cancer Screening Programme Managerial Guidelines.

[5] Dutta PK, Upadhyay A, Dutta M, Urmil AC, Thergaonkar MP, Ganguly SS (1990). A case control study of cancer cervix patients attending Command Hospital, Pune. Ind J Cancer.

[6] Caplash N, Sobti C (1999). Epidemiology of cervical cancer-a case control study on north indian population. Indian J Cancer.

[7] Prabhakar AK, Menon GR (1995). Age at marriage and cervical cancer incidence. Indian J Cancer.

